# The Potential and Limitations of Mobile Health and Insertable Cardiac Monitors in the Detection of Atrial Fibrillation in Cryptogenic Stroke Patients: Preliminary Results From the REMOTE Trial

**DOI:** 10.3389/fcvm.2022.848914

**Published:** 2022-04-13

**Authors:** Femke Wouters, Henri Gruwez, Julie Vranken, Dimitri Vanhaen, Bo Daelman, Ludovic Ernon, Dieter Mesotten, Pieter Vandervoort, David Verhaert

**Affiliations:** ^1^Limburg Clinical Research Center/Mobile Health Unit, Faculty of Medicine and Life Sciences, Hasselt University, Hasselt, Belgium; ^2^Department Future Health, Ziekenhuis Oost-Limburg, Genk, Belgium; ^3^Department of Cardiology, Ziekenhuis Oost-Limburg, Genk, Belgium; ^4^Department of Cardiovascular Sciences, University of Leuven, Leuven, Belgium; ^5^Department of Neurology, Ziekenhuis Oost-Limburg, Genk, Belgium; ^6^Department of Anesthesiology, Ziekenhuis Oost-Limburg, Genk, Belgium

**Keywords:** atrial fibrillation, cryptogenic stroke, insertable cardiac monitor (ICM), mobile health, cardiac rhythm monitoring

## Abstract

**Aim:**

This paper presents the preliminary results from the ongoing REMOTE trial. It aims to explore the opportunities and hurdles of using insertable cardiac monitors (ICMs) and photoplethysmography-based mobile health (PPG-based mHealth) using a smartphone or smartwatch to detect atrial fibrillation (AF) in cryptogenic stroke and transient ischemic attack (TIA) patients.

**Methods and Results:**

Cryptogenic stroke or TIA patients (*n* = 39) received an ICM to search for AF and were asked to use a blinded PPG-based mHealth application for 6 months simultaneously. They were randomized to smartphone or smartwatch monitoring. In total, 68,748 1-min recordings were performed using PPG-based mHealth. The number of mHealth recordings decreased significantly over time in both smartphone and smartwatch groups (*p* < 0.001 and *p* = 0.002, respectively). Insufficient signal quality was more frequently observed in smartwatch (43.3%) compared to smartphone recordings (17.8%, *p* < 0.001). However, when looking at the labeling of the mHealth recordings on a patient level, there was no significant difference in signal quality between both groups. Moreover, the use of a smartwatch resulted in significantly more 12-h periods (91.4%) that were clinically useful compared to smartphone users (84.8%) as they had at least one recording of sufficient signal quality. Simultaneously, continuous data was collected from the ICMs, resulting in approximately 6,660,000 min of data (i.e., almost a 100-fold increase compared to mHealth). The ICM algorithm detected AF and other cardiac arrhythmias in 10 and 19 patients, respectively. However, these were only confirmed after adjudication by the remote monitoring team in 1 (10%) and 5 (26.3%) patients, respectively. The confirmed AF was also detected by PPG-based mHealth.

**Conclusion:**

Based on the preliminary observations, our paper illustrates the potential as well as the limitations of PPG-based mHealth and ICMs to detect AF in cryptogenic stroke and TIA patients in four elements: (i) mHealth was able to detect AF in a patient in which AF was confirmed on the ICM; (ii) Even state-of-the-art ICMs yielded many false-positive AF registrations; (iii) Both mHealth and ICM still require physician revision; and (iv) Blinding of the mHealth results impairs compliance and motivation.

## Introduction

Cryptogenic stroke and transient ischemic attack (TIA) patients have no determined etiology at discharge and comprise about 35% of all ischemic stroke and TIA patients (1, 2). The most important risk factor for cryptogenic stroke is sub-clinical atrial fibrillation (AF) (3, 4). AF often remains undetected due to its often paroxysmal and asymptomatic nature (5). Mortality and stroke recurrence are twice as likely to occur in AF-related strokes compared to non-AF strokes. As such, they entail a higher burden on the patient and the healthcare system (6).

The risk of stroke in AF can be considerably reduced by oral anticoagulation (OAC) (6). However, according to current guidelines, AF should be documented for at least 30 s to warrant OAC therapy initiation (7). Insertable cardiac monitors (ICMs) are subcutaneously inserted and can reliably estimate the incidence and duration of AF episodes (i.e., AF burden) for up to 3 years (8). Moreover, the CRYSTAL-AF study demonstrated the superiority of ICMs vs. no prolonged rhythm monitoring (9). However, due to its invasive nature and high cost, the state-of-the-art ICM technology remains underutilized in the follow-up of cryptogenic stroke patients (9, 10).

Rapid progress in mobile technology supported the use of mobile devices in medical and public health practice, defined as mobile health (mHealth) (11). More specifically, novel smartphone and smartwatch applications have emerged as a non-invasive, inexpensive, and reliable alternative to detect AF (12, 13). In addition, mHealth allows the patient to perform numerous measurements in daily life without medical hardware. As such, mHealth could become a useful, long-term, less invasive add-on or alternative to ICMs in the detection of AF (14). Smartphone apps are very user-friendly as no additional device is necessary to detect AF. These apps (i.e., FibriCheck^®^ and Preventicus^®^ Heartbeats) use the photoplethysmography (PPG) principle (i.e., optical technique that detects blood volume changes) to perform spot-check rhythm monitoring (12, 13, 15, 16). On the other hand, smartwatches can detect AF by semi-continuous rhythm monitoring in an unobtrusive way using PPG (i.e., FibriCheck^®^ on a Fitbit^®^) (12, 17). Alternatively, electrodes implemented in the digital crown and back of the watch can be used to monitor the electrocardiogram (ECG) with point measurements (12). Several large companies (i.e., Apple^®^, Fitbit^®^, and Samsung^®^) have produced smartwatches that use both PPG and ECG (18–20). To our knowledge, PPG-based rhythm monitoring with a smartphone or smartwatch has not directly been compared to long-term continuous cardiac monitoring using an ICM in cryptogenic stroke or TIA patients. Although the digitization of healthcare was already in progress, the coronavirus 2019 (COVID-19) pandemic accelerated the shift toward mHealth and remote monitoring (21, 22). However, the ongoing REMOTE study encountered challenges still to be met. This paper elucidates the opportunities and limitations of using ICMs and PPG-based mHealth in cryptogenic stroke and TIA patients.

## Methods

This prospective, single-center, interventional, randomized trial compared the blinded use of PPG-based mHealth using a smartphone or smartwatch to guideline-recommended ICMs in cryptogenic stroke or TIA patients.

The study was started in September 2020, and the enrollment is ongoing. The protocol is in accordance with the Declaration of Helsinki and was approved by the medical ethics committees (i.e., Ziekenhuis Oost-Limburg, Genk, Belgium and Hasselt University, Hasselt, Belgium; 19/0093U). The study was registered at clinicaltrials.gov (NCT05006105).

### Study Population

**FIGURE 1:**
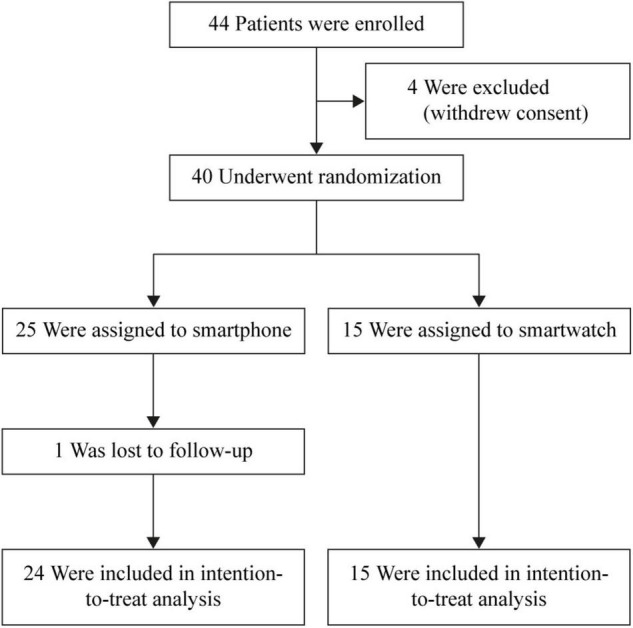
Flowchart of enrollment and randomization. ICM, insertable cardiac monitor.

The data presented in this paper were collected from cryptogenic stroke and TIA patients enrolled between September 2020 and 2021. The enrollment and randomization are presented in Figure 1. Inclusion and exclusion criteria are presented in the Supplementary Table 1. Inclusion criteria were diagnosis of cryptogenic ischemic stroke or TIA, the patient or its legal representative is willing to sign the informed consent, and the patient is 18 years or older. Exclusion criteria were history of AF or atrial flutter, life expectancy of less than 1 year, not qualified for ICM insertion, indication or contraindication for permanent OAC at enrollment, untreated hyperthyroidism, myocardial infarction or coronary bypass grafting less than 1 month before stroke onset, presence of patent foramen ovale (PFO) and it is or was an indication to start OAC according to the European Stroke Organization guidelines, inclusion in another clinical trial that would affect the objectives of this study, not able to understand the Dutch language, and the patient or partner is not in possession of a smartphone.

### Study Design

This study aimed to compare PPG-based mHealth and ICM-derived data in cryptogenic stroke or TIA patients. Patients used the mHealth tool (i.e., FibriCheck^®^, Qompium NV, Hasselt, Belgium) for 6 months starting from the day of ICM insertion. The subjects were randomized in a 1:1 manner to use either a smartphone or smartwatch to perform PPG-based rhythm monitoring. Patients in the smartphone group were asked to perform 2 1-min measurements each day and in case of symptoms. Subjects in the smartwatch group were asked to continuously wear the smartwatch, which performed semi-continuous measurements (i.e., automatic recording of 1 min, every 3 min). These patients were also allowed to perform recordings using their smartphones. The adjudication of the mHealth recordings was based on the mHealth algorithm followed by a Qompium physician overreading in case of irregularities detected by the FibriCheck^®^ algorithm. The results of the PPG-based rhythm monitoring recordings were blinded for both patient and caregiver during the study. Remote monitoring of irregularities detected by the ICM algorithm was conducted every weekday by a dedicated remote monitoring team, according to the usual clinical care. Labeling of the ICM data was performed in two steps. First, the ICM device algorithm identified episodes of heart rhythm irregularity potentially consistent with AF or other arrhythmias. Subsequently, these episodes were adjudicated by a dedicated remote monitoring team.

To determine the adherence to the protocol in the smartphone group, two parameters were specified. Compliance describes to what extent the patient performed the expected number of recordings. It was calculated as the total number of performed spot-checks divided by the total number of recommended spot-checks (i.e., two measurements each day during 180 days). Motivation gives more information about the regularity or consistency with which the recordings were performed. It was calculated as the number of days with at least two daily spot-checks divided by the number of days on which the application should be used (i.e., 180 days when a patient completed the 6-month follow-up or fewer in case follow-up was still ongoing). As there was no minimal recommended number of measurements per day for the smartwatch group, the compliance and motivation were not calculated for these patients. Therefore, the number of recordings per day was calculated.

### Data Collection

Demographic and clinical characteristics of the subjects were obtained from the electronic medical record (HIX, Chipsoft, Netherlands) and the device dashboards (Biotronik, Germany; Medtronic, Ireland; Qompium, Belgium) and collected in the electronic case report form (Castor EDC, Netherlands).

### Statistical Analysis

Sample size calculations were performed by CenStat (Hasselt University). Since we did not expect an increased AF detection rate by mHealth compared to ICMs, non-inferiority testing was chosen. Based on literature, we assumed an AF detection rate after 6 months of 20% and 15% by ICM and mHealth, respectively (9, 23, 24). The non-inferiority margin was set at 0.07 to achieve an improved AF detection compared to a 7-day Holter (25). Since a control method (i.e., ICM) was compared with two other methods (i.e., PPG-based mHealth on smartphone and smartwatch), a Bonferroni-correction was applied. A total sample size of 225 patients is expected to achieve 80% power, including a drop-out rate of 10%.

Data analysis was performed using the Statistical Package for Social Sciences release 28.0 (IBM^®^ SPSS^®^ Inc., Chicago, IL, United States). A *p* < 0.05 was considered statistically significant unless specified otherwise. The Shapiro–Wilk statistic assessed the normality of the continuous data. The continuous variables are presented as mean ± standard deviation (SD) or median and interquartile range (IQR) when appropriate. Discrete variables are presented as absolute numbers and percentages (%). An intention-to-treat analysis was used to compare demographic and clinical characteristics between the smartphone and smartwatch groups. These analyses were performed using the Pearson’s Chi-Square test, Fisher’s exact test, Mann–Whitney U test, Likelihood Ratio, or independent *t*-test. The mHealth measurements were analyzed using Pearson’s Chi-Square test and Fisher’s exact test, based on which device was used to perform the recording. Finally, the Friedman test and *post-hoc* Sign test with Bonferroni correction applied were performed to compare the compliance, motivation, and the number of recordings performed over time.

## Results

Forty-four cryptogenic stroke or TIA patients were enrolled. Due to the COVID-19 pandemic, four patients withdrew consent. One patient was lost to follow-up. Therefore, 39 subjects were considered in the analyses (Figure 1).

### Study Population

The demographic and clinical characteristics of the included subjects are presented in Table 1. There were no significant differences in the demographic characteristics between the smartphone and the smartwatch groups.

**TABLE 1:** Demographic and clinical characteristics.

Characteristic	Smartphone group (*n* = 24)	Smartwatch group (*n* = 15)
Age, years	63.0 ± 12.6	68.7 ± 9.3
Sex, male	19 (79.2%)	8 (53.3%)
BMI, kg/m^2^	26.9 [24.3 – 29.2]	28.4 [23.9 – 30.1]
PFO	7 (29.2%)	5 (33.3%)
**Index event**		
Stroke	15 (62.5%)	9 (60.0%)
TIA	9 (37.5%)	6 (40.0%)
Prior stroke	0 (0.0%)	3 (20.0%)
Prior TIA	3 (12.5%)	0 (0.0%)
Score on NIH Stroke Scale*	1 [0.5 – 3]	1 [0 – 2]
Hypertension	17 (70.8%)	9 (60.0%)
Diabetes	3 (12.5%)	2 (13.3%)
Hypercholesterolemia	14 (58.3%)	7 (46.7%)
Current smoker	9 (37.5%)	3 (20.0%)
CHA_2_DS_2_-VASc score**	4 [3 – 5]	4 [3 – 5]
Mean time between index event and ICM insertion, days	77.5 [62.0 – 112.3]	88.0 [63.0 – 144.0]

BMI, body mass index; ICM, insertable cardiac monitor; PFO, patent foramen ovale; TIA, transient ischemic attack. *Score on National Institutes of Health Stroke Scale ranges from 0 to 42; higher score indicates more severe neurologic deficits. **CHA_2_DS_2_-VASc score ranges from 0 to 9; higher score indicates an increased risk of stroke.

### Arrhythmia Detection and Annotation of Insertable Cardiac Monitors

The ICMs collected continuous data, resulting in approximately 111,000 h, or 6,660,000 min of data. The ICM algorithm detected 259 potential AF episodes in 10 different patients. After adjudication, these episodes were labeled as AF (5, 1.9%, all in 1 patient), sinus rhythm (200, 77.2%, in 8 patients), ectopic beats (40, 15.4%, in 3 patients), oversensing (1, 0.4%), or noise (2, 0.8%, in 1 patient); 11 episodes (4.3%, in 2 patients) were not labeled.

Besides AF, the ICM algorithm also identified other relevant arrhythmias such as pause (221, 7.4%), tachycardia or tachyarrhythmia (tachy, 83, 2.8%), atrial tachycardia (AT, 2,653, 88.8%), high ventricular rate (14, 0.5%), and bradycardia or bradyarrhythmia (brady, 17, 0.6%) episodes. The remote monitoring team adjudicated only 349 of these other relevant arrhythmia episodes. The labeling of the arrhythmias by the ICM device algorithm and their adjudication by the remote monitoring team as either disapproved (i.e., the algorithm-generated label was inappropriate) or confirmed (i.e., the label was appropriate) is presented per patient in Table 2.

**TABLE 2:** Number of patients with a cardiac arrhythmia detected by insertable cardiac monitor and labeling of the mHealth recordings per patient performed with smartphone or smartwatch.

Insertable cardiac monitor		

Label	Disapproved	Confirmed
Atrial fibrillation (*n* = 10)	9 (90.0%)	1 (10.0%)
Pause (*n* = 8)	6 (75.0%)	2 (25.0%)
Tachycardia/tachyarrhythmia (*n* = 7)	6 (85.7%)	1 (14.3%)
Atrial tachycardia (*n* = 1)	1 (100%)	0 (0.0%)
High ventricular rate (*n* = 2)	1 (50.0%)	1 (50.0%)
Bradycardia/bradyarrhythmia (*n* = 1)	0 (0.0%)	1 (100%)
**Photoplethysmography-based mobile health**		

**Label**	**Smartphone(*n* = 39)**	**Smartwatch(*n* = 15)**

Sinus rhythm	38 (97.4%)	15 (100%)
Insufficient signal quality	32 (82.1%)	14 (93.3%)
Other arrhythmias	16 (41.0%)	14 (93.3%)
Suspicious for atrial fibrillation	4 (10.3%)	6 (40.0%)

*Data presented as n (%).*

### Arrhythmia Detection and Annotation of mHealth

The subjects performed a total of 68,748 1-min recordings using PPG-based mHealth; 5,030 (7.3%) using a smartphone, and 63,718 (92.7%) using a smartwatch. All patients randomized to the smartwatch group also performed recordings on their smartphone, either temporary or permanent. More than half of the subjects (*n* = 26) reported symptoms during 350 (0.5%) recordings. The mHealth recordings were labeled sinus rhythm (*n* = 38,819), insufficient signal quality (*n* = 28,509), suspicious for AF (*n* = 101), or other arrhythmias (i.e., ectopic beats) (*n* = 1,315), presented per patient in Table 2. Four measurements showed no result. There was a significant difference in the mHealth recordings’ labeling between the smartphone and smartwatch groups (*p* < 0.001) for all labels (i.e., sinus rhythm, insufficient signal quality, suspicious for AF, and other arrhythmias). More specifically, sinus rhythm, suspicious for AF, and other arrhythmias were more present in the smartphone group, whereas insufficient signal quality was more prevalent in smartwatch users. However, when looking at the labeling of the mHealth recordings on a patient level, there was only a significant difference for the labels suspicious for AF and other arrhythmias between both groups (*p* = 0.02 and *p* < 0.001, respectively). The patient in which AF was detected and adjudicated as such by the ICM, also performed mHealth recordings using a smartphone. These recordings were labeled as suspicious for AF.

For both smartphone and smartwatch groups, there were 4,809 periods of 12 h in which at least one measurement was performed using mHealth. In 4,133 (85.9%) of these periods, at least 1 recording had sufficient signal quality to be evaluated and was, therefore, clinically useful. There was a statistically significant difference between smartphone- and smartwatch-performed recordings (*p* < 0.001). Using a smartphone, 3,362 (84.8%) out of the 3,965 12-h periods had at least 1 measurement performed with sufficient signal quality. On the other hand, 771 (91.4%) out of the 844 12-h periods were clinically useful when using a smartwatch.

### Compliance, Motivation, and Number of Measurements Performed With mHealth

**FIGURE 2:**
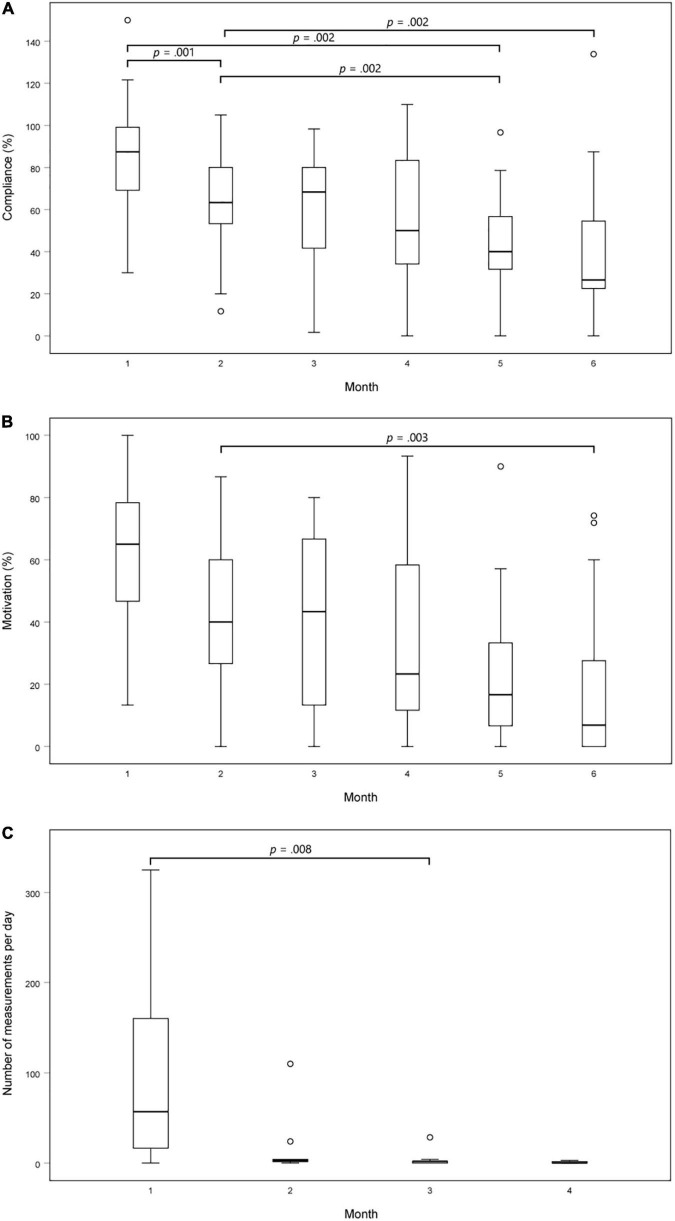
Adherence to the protocol over time. **(A)** Compliance of using PPG-based mHealth on a smartphone over time. This was calculated as the total number of spot-checks performed divided by the total number of recommended spot-checks (i.e., two measurements each day); **(B)** Motivation of using PPG-based mHealth on a smartphone over time. This was calculated as the number of days with at least two daily spot-checks divided by the number of days on which the application should be used; **(C)** Number of recordings performed per day using mHealth on a smartwatch over time. *P*-values were calculated using a Friedman test and *post-hoc* Sign test with Bonferroni correction applied.

The compliance and motivation of using mHealth were determined for the patients in the smartphone group. This resulted in a compliance of 60.4% ± 23.0% and a motivation of 40.6% ± 22.4%. Both compliance and motivation decreased after the 1st month (*p* < 0.001) (Figures 2A,B). *Post-hoc* analysis with Sign test was conducted with a Bonferroni correction applied, resulting in a significance level set at *p* < 0.0033 to compare differences between the different months.

The total monitoring duration of all patients in the smartwatch group was 1,123 days. On 357 (31.8%) days, no measurements were performed. In theory, 480 out of 1,440 min were expected to be monitored using PPG-based mHealth daily. However, only a median of 19.5 [3 – 146.5] minutes per day were monitored. Due to technical issues, 6 (40.0%) patients eventually used their smartphones to perform the recordings, resulting in a reduction of performed measurements per day. As such, there was a statistically significant difference (*p* = 0.002) over the months in the median number of measurements performed in the smartwatch group (Figure 2C). *Post-hoc* analysis with Sign test was conducted with a Bonferroni correction applied, resulting in a significance level set at *p* < 0.0083. Median [IQR] number of measurements per day in month 1, 2, 3, and 4 were 57 [2 – 178], 3 [1 – 4], 2 [0 – 3], and 0 [0 – 2], respectively. There was a statistically significant reduction in the number of measurements performed in the 3rd month compared to the 1st month (*p* = 0.008).

## Discussion

This paper presents the opportunities and limitations of using PPG-based mHealth compared to ICMs in cryptogenic stroke and TIA patients.

### Both Insertable Cardiac Monitor and Photoplethysmography-Based mHealth Detected Atrial Fibrillation in the Same Patient

Only five AF episodes detected by the ICM algorithm in one patient were confirmed after physician revision. The confirmed AF episodes were also detected by PPG-based mHealth on a smartphone. The number of patients in which AF was detected, is lower than expected. However, not all patients have already been monitored for 6 months. Overall, the median time between index event and ICM insertion was 81 (62 – 117) days. This is two to three times longer compared to other studies (9, 23). In real-life, this intermediate period could be bridged by using mHealth applications.

### State-of-the-Art Insertable Cardiac Monitors Yield False-Positive Atrial Fibrillation Registrations, and Together With mHealth Requires Adjudication

Remarkable was the substantial proportion of false-positive AF episodes reported by the state-of-the-art ICMs. After revision by dedicated remote monitoring nurses or cardiologists, most of these retained “AF episodes” were redefined as sinus rhythm, ectopic beat, noise, or oversensing. As such, 259 AF episodes detected by the ICM had to be revised by a physician. On the other hand, 101 PPG-based mHealth recordings detected AF and could require a second revision by a physician. This was also the case for other non-AF arrhythmias selected by the ICM algorithm, likewise, often judged as inappropriately labeled. As such, 2,988 other arrhythmias were detected by the ICM and required physician revision. PPG-based mHealth detected 1,315 other arrhythmias that could demand a second revision. Consequently, ICMs require an even higher workload to revise cardiac rhythm irregularities compared to PPG-based mHealth.

### Differences Between Smartphones and Smartwatches

The proportions of the different labels were significantly different between smartphone- and smartwatch-performed recordings. More arrhythmias were detected in the smartphone group compared to the smartwatch group. This might be because recordings were more often performed when patients experienced symptoms, whereas while using a smartwatch, most recordings were performed unconsciously. The most interesting finding was that insufficient signal quality was significantly more present in recordings performed with a smartwatch compared to a smartphone. This result may be explained because recordings performed with a smartwatch (i.e., passive measurements) are more sensitive to motion artifacts since patients are mostly unaware when a measurement is being recorded. In contrast, the patient has to perform a recording using a smartphone actively, and thus, is more likely to remain still during the measurement.

However, when looking at the labeling of the mHealth recordings on a patient level, there was no significant difference in signal quality between both groups. Furthermore, to establish if this would impair the physician to evaluate the heart rhythm of a patient twice daily, the number of 12-h periods with at least one recording of sufficient quality was determined. This demonstrated that the use of a smartwatch resulted in significantly more 12-h periods that were clinically useful compared to smartphone users. This is due to the increased amount of measurements performed with a smartwatch compared to a smartphone. As such, the chances of having at least one valuable recording are higher compared to when only two measurements are performed using a smartphone.

### Blinding of the mHealth Results Impairs Compliance and Motivation

Thus far, there was limited information about long-term compliance and motivation of PPG-based mHealth prescribed in a cryptogenic stroke or TIA population. However, it has been demonstrated to generate good compliance and motivation in other populations (24, 26). Since we could not compute the compliance and motivation for the smartwatch group, this group cannot be compared with the smartphone group. However, it is noteworthy that this adherence to the protocol reduced significantly over time in both groups. Overall, patients in the smartphone group became less compliant and motivated to perform two measurements each day over time. This can be a result of the blinding of the measurements’ results. Lack of feedback might impair the patient’s motivation to perform two recordings daily.

### Strengths and Limitations of mHealth Using a Smartwatch

The emergence of novel medical smartphone and smartwatch applications underscores the value of mHealth in a hyperconnected digital world and exemplifies the digital transformation in healthcare (13, 27, 28). The added value of PPG-based mHealth performed on smartwatches is the possibility to perform semi-continuous measurements, approximating the continuous nature of ICMs. In this study, a recording was performed automatically every 3 min. In theory, this results in 480 measurements performed each day. On average, only a mere 20 recordings were performed daily. Similar to the smartphone group, the number of measurements performed per day decreased significantly over time. This is because patients did not continuously wear the watch as it needed to recharge almost daily due to this strenuous measurement schedule. Moreover, some technical problems occurred in this group. An inactive measurement schedule caused most technical issues. Another but less prevalent technical issue was a disruption in the Bluetooth connection between the watch and the phone. As such, the recordings could not be uploaded to the cloud. Since only a limited number of recordings could be saved on the watch, this might have led to data loss.

Besides technology issues, a reduced number of measurements could also be due to the operator. In this study, cryptogenic stroke or TIA patients were included. Memory dysfunction is often present in this population (29). However, patients received daily reminders to perform recordings or wear their watch.

As a result, the number of recordings performed per day in the smartwatch group decreased considerably over time compared to what was expected. This could theoretically affect the sensitivity of the smartwatch, particularly for detecting short-lived episodes of AF in paroxysmal AF patients. Nevertheless, PPG-based mHealth used in this study was programmed to identify AF when the duration exceeds 30 s. ICMs, in contrast, require at least 2 min of AF to minimize false positives (12, 23). On the other hand, there is no consensus on the threshold of AF episode durations that are clinically relevant, especially in stroke patients (30). However, a study performed by Singer et al. confirmed the direct and transient association between AF and stroke while using an AF duration threshold of 5.5 h. Furthermore, they found that AF episodes with a duration of more than 23 h were associated with the most significant increased stroke risk (31). Therefore, two discrete mHealth spot-check recordings per day using a smartphone or smartwatch should be sufficient to detect clinically relevant AF episodes.

### Study Limitations

A head-to-head comparison between PPG-based mHealth and ICMs in the detection of AF could not be described due to a limited amount of data. Therefore, these preliminary data analyses focused on detecting the different arrhythmias and their adjudication by the remote monitoring team. A limitation of this study is the blinding of the PPG-based mHealth results for both patient and caregiver. Mainly because it diminishes the compliance and motivation of patients to perform the recommended number of recordings per day. However, this was necessary to ensure that all clinical decision-making was solely based on the findings from the state-of-the-art ICMs, as recommended by current guidelines. Another limitation to the widespread implementation of mHealth is the fact that particularly smartwatch technology has not yet been widely adopted, especially not in the older population. This would require a care system in which hospitals provide a smartwatch to either bridge the period between index event and ICM insertion or replace ICMs in those who refuse to have an ICM inserted. Finally, a reduction in stroke recurrence after an optimized AF detection strategy is yet still to be demonstrated (23). However, it is known that stroke recurrence is twice as likely to occur in AF-related strokes (6). Furthermore, several studies have already suggested a decrease in stroke recurrence risk in cryptogenic stroke patients who received OAC after AF detection on ICM (9, 32). Nevertheless, there are still many unanswered questions about the clinical relevance of short AF episodes and whether using PPG-based mHealth might be sufficient to detect longer AF episodes.

## Conclusion

This paper indicated the potential of PPG-based mHealth using smartphones and smartwatches to detect AF in a cryptogenic stroke and TIA population while presenting the constraints from both ICM and PPG-based mHealth on smartwatches. PPG-based mHealth was able to detect AF in a patient in which AF was confirmed on the ICM. However, even state-of-the-art ICMs yielded many false-positive AF registrations. Consequently, both mHealth and ICMs still require deliberation by trained nurses or cardiologists. Besides technical issues, blinded mHealth also suffered from a reduction in compliance and motivation with long-term use. More data is necessary to compare the results of both cardiac monitoring methods.

## Data Availability Statement

The raw data supporting the conclusions of this article will be made available by the authors, without undue reservation.

## Ethics Statement

The studies involving human participants were reviewed and approved by the Comité Medische Ethiek, Ziekenhuis Oost-Limburg, and Hasselt University. The patients/participants provided their written informed consent to participate in this study.

## Author Contributions

FW and DVa were involved in the data collection. FW performed the statistical analysis and drafted this manuscript. All authors read, reviewed, and edited the manuscript and contributed to the article and approved the submitted version.

## Conflict of Interest

The authors declare that the research was conducted in the absence of any commercial or financial relationships that could be construed as a potential conflict of interest. The handling editor DD declared a past collaboration with the authors HG, PV, and DVe.

## Publisher’s Note

All claims expressed in this article are solely those of the authors and do not necessarily represent those of their affiliated organizations, or those of the publisher, the editors and the reviewers. Any product that may be evaluated in this article, or claim that may be made by its manufacturer, is not guaranteed or endorsed by the publisher.
