# Supplementary material for: The Potential and Limitations of Mobile Health and Insertable Cardiac Monitors in the Detection of Atrial Fibrillation in Cryptogenic Stroke Patients: Preliminary Results From the REMOTE Trial
Source: Front Cardiovasc Med. 2022 Apr 13;9:848914. doi: 10.3389/fcvm.2022.848914 (PMC9043805; doi:10.3389/fcvm.2022.848914)
Supplement: Supplementary file 1 [file Table_1.DOCX]

Supplementary Material

Table 1. Overview of the inclusion and exclusion criteria.

| **Inclusion criteria** | **Exclusion criteria** |
| --- | --- |
| Diagnosis of cryptogenic ischemic stroke or TIA  The patient or its legal representative is willing to sign the informed consent  18 years or older | History of AF or atrial flutter  Life expectancy of less than one year  Not qualified for ILR insertion  Indication or contraindication for permanent OAC at enrolment  Untreated hyperthyroidism  Myocardial infarction or coronary bypass grafting less than one month before the stroke onset  Presence of PFO and it is or was an indication to start OAC according to the European Stroke Organization guidelines  Inclusion in another clinical trial that will affect the objectives of this study  Not able to understand the Dutch language  Patient or partner not in possession of a smartphone |

*AF, atrial fibrillation; ILR, insertable loop recorder; OAC, oral anticoagulation; PFO, patent foramen ovale; TIA, transient ischemic attack.*
